# Anesthetic consideration for airway management of patients undergoing endoscopic fibrin glue treatment of tracheobronchial rupture: a case series of three patients

**DOI:** 10.1186/s13741-018-0111-x

**Published:** 2018-12-17

**Authors:** Vincenzo Pota, Pasquale Sansone, Alfonso Fiorelli, Maria Beatrice Passavanti, Mauro D’Amora, Paola Vosa, Elena Bignami, Maria Caterina Pace, Caterina Aurilio

**Affiliations:** 1 Anaesthesia and Intensive Care Unit, Department of Women, Children, General and Specialistic Surgery, L. Vanvitelli University of Campania, Naples, Italy; 2 Thoracic Surgery Unit, L. Vanvitelli University of Campania, Naples, Italy; 3 Anaesthesia and Intensive Care Unit, San Carlo Hospital, Potenza, Italy; 4 Anaesthesia and Intensive Care Unit, A.O.R.N. Santobono Pausillipon, Naples, Italy; 5 Anaesthesia, Intensive Care and Pain Medicine, Department of Medicine and Surgery, University of Parma, Parma, Italy

**Keywords:** Tracheobronchial ruptures, Airway management, Case report

## Abstract

Tracheobronchial ruptures are very severe and life-threatening injuries. The origin of such airway damage is trauma or an iatrogenic event. Last year, we operated on three different cases of tracheal ruptures using endobronchial suture with three different airway management. We exposed the description of three different techniques to manage the airway during an endoscopic suture of tracheobronchial rupture with fibrin glue (laryngeal mask, orotracheal tube positioned distally the lesion, one lung ventilation with a small size single tube). Using this kind of technique, we have obtained a protective ventilation on tracheal rupture, a rapid healing, and fast recovery of spontaneous breathing.

## Introduction

Tracheobronchial ruptures are very severe and life-threatening injuries. The origin of such airway damage is trauma or an iatrogenic event. Most iatrogenic tracheobronchial lesions are due to tracheal intubation. Other rarer causes are percutaneous tracheostomy, lesions related to stiff bronchoscopy, mechanical ventilation, thyroid surgery, esophageal surgery, and lung surgery (Gil et al. 2016). A team approach to tracheal rupture intervention that involves the thoracic surgeon intensivist/anesthesiologist is very important. The intensivist/anesthesiologist must have expertise both in a difficult airway and in lung surgery. The first problem is choosing in between thoracotomy and an endoscopic approach. The Thoracic Surgery Unit of University of Campania “L. Vanvitelli” has particular expertise in endoscopic suture by fibrin glue of tracheobronchial rupture. Last year, we operated on three different cases of tracheal rupture using endobronchial suture with three different airway management devices: (i) laryngeal mask (LMA), (ii) an orotracheal tube positioned distally the lesion, (iii) and one lung ventilation with a small size single tube. The main problem for the anesthesiologist is the airway management, ventilation, and the fast recovery of spontaneous breathing.

## Case 1

**Fig. 1:**
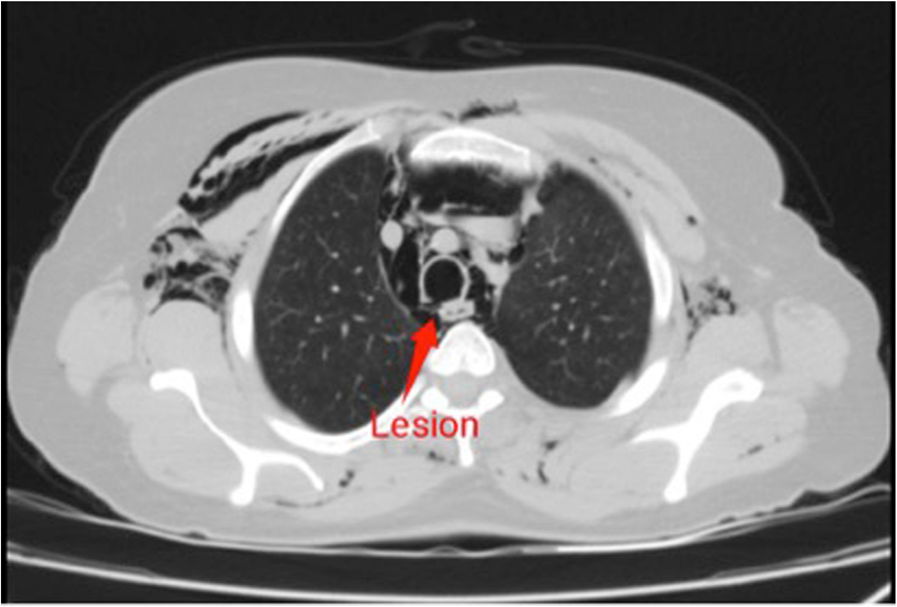
CT scan case 1 tracheal rupture

**Fig. 2:**
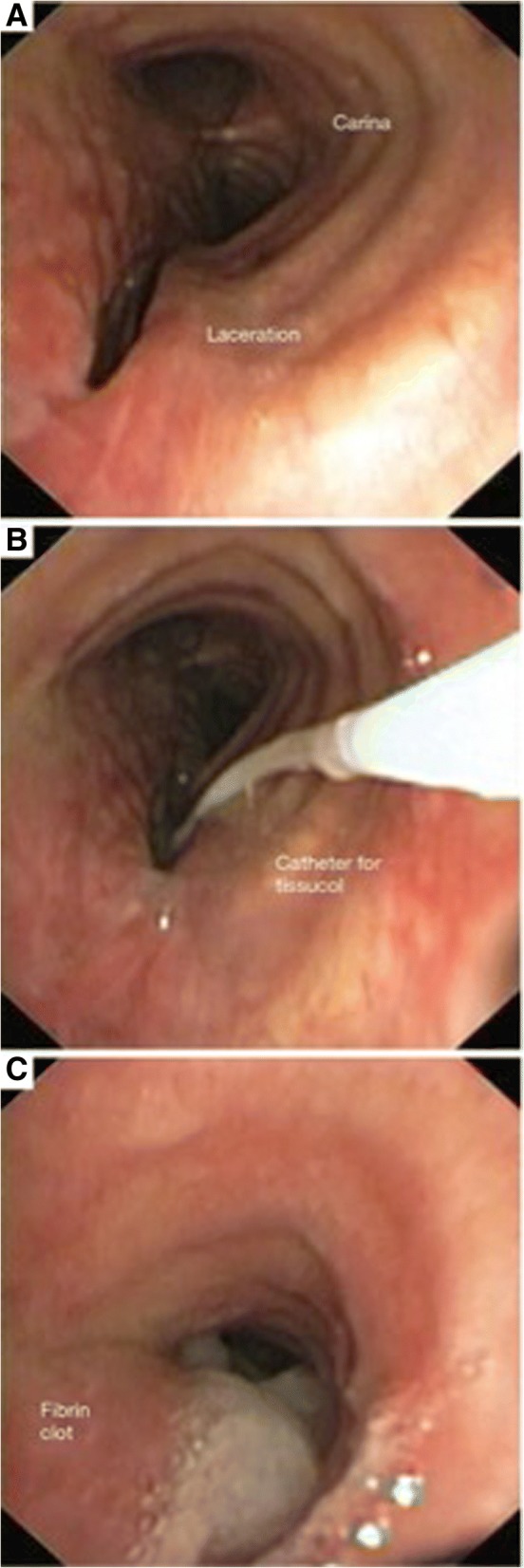
**a**–**c** Case 2—bronchoscopic suture by fibrin glue

A 40-year-old woman, 90 kg, was admitted to the Intensive Care Unit of University of Campania “L. Vanvitelli” from an outlying hospital because of an intubation-related tracheal lesion. The intubation has been necessary for the removal of right vocal cord using direct suspension microlaringoscopy (SDML) with CO_2_ laser. The patient showed severe subcutaneous emphysema extended from the chest up to the neck and head. She was affected by dyspnea, with 50% inspired oxygen through the Venturi mask. Blood pressure (BP) was 150/95 mmHg, mean arterial pressure (MAP) 113 mmHg, heart rate (HR) was 120 bpm, and peripheral oxygen saturation (SpO2) was 93%. The neck and chest CT (computerized axial tomography) scan showed “a 4-cm length lesion into the right posterolateral wall of the trachea in correspondence with the fourth dorsal vertebra, about 37 mm from carena; pneumomediastinum; diffuse signs of subcutaneous and perimuscular emphysema were visible, from the chest till to the neck to the head; and normal expansion of lung areas.” (Fig. 1). The patient was, immediately, carried to the operating theater for a diagnostic bronchoscopy and for the endoscopic suture with fibrin glue. We decided to manage the airway using an LMA positioned in deep sedation. Sedation was induced using propofol 4 mg/kg/h and fentanyl 1 μg/kg. We also administered 0.5 mg/kg of intravenous lidocaine to blunt laryngeal reflexes. We conducted the anesthesia by mean of spontaneous breathing with oxygen supplement throughout manual breathing circuit. The patient was placed in extended neck position. The blood pressure monitoring was executed by left radial artery catheterization and pulse oximetry on the other side. Heart rate and end expiratory CO_2_ (ETCO_2_) were also monitored. The flexible bronchoscope was introduced through the LMA (Fig. 2). Intraoperative monitoring: the thoracic surgeon first of all confirmed the tracheal lesion and then sutured it with 6 ml of fibrin glue. The procedure lasted about 30 min. During the procedure, all the hemodynamic parameters were stable. At the end, the patient was awakened in the operating room and moved to the Intensive Care Unit spontaneously breathing with 50% inspired oxygen through the Venturi mask. Forty-eight hours after the surgery, a chest and neck CT scan was made. It showed the resolution of pneumomediastinum and subcutaneous emphysema, and so the patient was transferred to the thoracic surgery ward and 72 h after the surgery she was discharged. She came back 15 days after the surgery and for a diagnostic flexible bronchoscopy that showed a complete resolution of the tracheal rupture.

## Case 2

A 53-year-old woman, 125 kg weight, 175 cm tall, body mass index (BMI) 40.8 kg/m^2^, was admitted to the Intensive Care Unit of University of Campania “L. Vanvitelli” from a nearby hospital because of an iatrogenic intubation-related tracheal lesion. The intubation has been necessary in order to perform anesthesia for a laparoscopic sleeve gastrectomy. The patient was affected also by hypertension and hypercholesterolemia. She arrived with an orotracheal tube (size 7.0 mm reinforced) and in assisted ventilation through manual breathing circuit. She presented a severe subcutaneous emphysema from the chest till to the neck to the head. The hemodynamic parameters were not too stable (BP 180/100 mmHg, MAP 127 mmHg, HR 130 bpm, SpO2 97%). A diagnostic fibrobronchoscopy was immediately performed. It showed a 2-cm lesion of pars membranacea in subglottic region. During fibrobronchoscopy, the orotracheal tube was changed with a 6.0-mm-size tube, positioned distally the lesion and before the carena. A chest radiograph was made, and pneumomediastinum and subcutaneous emphysema were confirmed. The patient was thus carried to the operating room. We decided to anesthetize the patient with propofol 6 mg/kg/h and fentanyl 2 μg/kg. We also administered 0.5 mg/kg of intravenous lidocaine to blunt laryngeal reflexes. The neuromuscular blockage was achieved with rocuronium bromide 0.6 mg/kg. During the procedure, protective mechanical ventilation was set up (tidal volume 6 ml/kg, positive end expiratory pressure (Peep) 5 mmHg, FiO_2_ 50% respiratory rate 15). The patient was placed in extended neck position. The blood pressure monitoring was done by left radial artery catheterization and pulse oximetry on the other side. Heart rate and ETCO_2_ were also monitored. The flexible bronchoscope was introduced next to the lesion and before the tube cuff. The thoracic surgeon first of all confirmed the tracheal lesion, and then, he sutured it with 5 ml of fibrin glue. At the end of the surgery, the patient was awakened and extubated and then she was carried to the Intensive Care Unit for postoperative monitoring in spontaneous breathing with 50% inspired oxygen through the Venturi mask. Forty-eight hours after the surgery, esophageal X-ray was taken to exclude an esophageal lesion and to check the sleeve gastrectomy and eventually the nasogastric tube was removed. Seventy-two hours after the surgery, there was a notable reduction of subcutaneous emphysema and good hemodynamics. Consequently, the patient was moved to the thoracic surgery ward.

## Case 3

A 63-year-old woman, weighing 60 kg, 165 cm tall, with BMI of 22 kg/m^2^ was admitted to the Intensive Care Unit of University of Campania “L. Vanvitelli” from a regional hospital because of an iatrogenic tracheal lesion probably due to the removal of a neoplasm that was involving the epiglottis and vocal cords. At the end of the surgery, during the patient’s awakening, hemoptysis showed up with dyspnea and a fibrobronchoscopy had to be made that showed a lesion on the right bronchus. Excluding the right lung, a double-lumen tube (Robertshaw 37F) was positioned in order to reduce the air leak and protect the airway from bleeding and then the patient was carried to the Intensive Care Unit of University of Campania “L. Vanvitelli”. The patient was suffering from hypertension. She was affected by subcutaneous emphysema from the chest to the neck and the head; thus, a right chest drainage was positioned because of pneumothorax. The blood gas analysis showed respiratory acidosis with hypoxia (partial pressure of oxygen (PaO_2_) 75 mmHg) and hypercapnia (partial pressure of carbon dioxide (PCO_2_) 60 mmHg). The patient was connected to a mechanical ventilator (FiO_2_ 65%, Peep 5, tidal volume 4 ml/kg, inspiratory/expiratory ratio 1/2.5). A flexible fibrobronchoscopy was taken that showed a lesion of the right bronchus 4 cm distal the carena. A chest X-ray demonstrated emphysema of the chest wall and neck, pneumomediastinum, and bilateral pleural effusion. The patient was transferred to the operating room in order to proceed with the operative bronchoscopy. We decided to manage the airway by removing the double-lumen tube and positioning a small size single lumen tube (5.5) into the left bronchus in anesthesia with propofol 6 mg/kg/h and fentanyl 2 μg/kg. The neuromuscular blockage was achieved by rocuronium bromide 0.6 mg/kg. We also administered 0.5 mg/kg of intravenous lidocaine to blunt laryngeal reflexes. During the procedure, the mechanical ventilation was started (tidal volume 4 ml/kg, Peep 5, FiO_2_ 50%, respiratory rate 18). The flexible bronchoscope was introduced beside the orotracheal tube next to the lesion, and the thoracic surgeon sutured the bronchus lesion with 5 ml of fibrin glue. After the surgery, the patient was carried to the intensive care unit without changing either the tube or the mechanical ventilation (tidal volume 4 ml/kg, Peep 5 mmHg, FiO_2_ 50%, respiratory rate 18). Twelve hours after the surgery, a consultation with an otolaryngologist was asked for and a flexible videolaryngoscopy was taken which both confirmed edema and hyperemia of the larynx, glottis, and subglottic region. The extubation was not considered safe, the single-lung ventilation was no longer acceptable, and the normal double-lung ventilation could be detrimental to the bronchus suture. So we decided to practice a percutaneous tracheostomy (single-dilator percutaneous tracheostomy Ciaglia techniques), and at the end of the procedure, the mechanical ventilatory weaning both in analgosedation with remifentanil and in assisted ventilation (Peep 0, pressure support 8, FiO_2_ 0, 45 tidal volume 500, respiratory rate 16) was carried out. Twenty-four hours after the surgery, the patient was freed from mechanical ventilation and put in spontaneous breathing by tracheostomy with oxygen supplement 5 l/min. Ninety-six hours after the surgery, there was a notable reduction of subcutaneous emphysema and good hemodynamics and so the patient was moved to the thoracic surgery ward. Eighteen days after the surgery, a new consultation with the otolaryngologist and a flexible videolaryngoscopy were carried out that showed the complete resolution of edema; hyperemia of the larynx, glottis, and subglottic region; and normal mobilization of the vocal cords and so the tracheostomy tube was removed, and the day after, the patient was discharged from the hospital.

## Discussion

One of the most important aspects that is emerging from literature and from our case series is the necessity of continuous dialogue between thoracic surgeon and anesthesiologist/intensivists in order to plan together the therapeutic approach and so the best airway management. It should be desirable to create a multi-disciplinary tracheal rupture team, involving thoracic surgeons, intensivists, radiologists, and otolaryngologists with special expertise in those pathologies. The most frequent cause of tracheal rupture is the orotracheal intubation with an incidence that ranges between 0.05 and 0.3% (Harenberg et al. 2010; Iwanczuk et al. 2008). The risk factors that increase this incidence are urgent and emergency intubation, out of hospital intubation, double-lumen tube intubation, and unanticipated difficult airway (Schneider et al. 2009).

From a 1999 database on lesions due to orotracheal intubation emerges that lesions can interest the nose (5%), temporo-mandibular joint (10%), pharynx (19%), larynx (33%), tracheobronchial airway (15%), and esophagus (18%). It is interesting to notice that 69% of tracheobronchial lesions related to orotracheal intubation occur during an intubation non-referred as difficult (Domino et al. 1999). Another analysis, instead, showed that the incidence of airway lesions during non-difficult intubation and elective surgery is about 9% (Cheney et al. 1991). An interesting study published in 2016 examined 24 patients with iatrogenic lesions of the trachea or bronchus: in 16 patients (66%), lesions were certainly related to tracheal intubation. Seven patients (7/16) presented lesions in the upper half of the trachea, 9 patients (9/16) in the lower half, and in particular 4 in the right main bronchus and 2 in the left main bronchus. Only 3 patients presented lesions due to urgent intubation; all the rest (13) were complication of elective surgery and intubation (Gil et al. 2016). Moreover, in the same study, 12 patients had an inferior to 3-cm-long lesion, 10 patients had a lesion that extended from 3 to 5 cm, and 2 patients an above 8-cm-length lesion. The risk factors of tracheal lesions can be divided into patient characteristics (tracheal anomalies, tracheomalacia, old age, women gender), the anesthesiologist’s experience (correct tube positioning, several attempts of intubation, tube repositioning without deflating the cuff), tube (not correct size, double-lumen tube), and intubation technique (non-correct use of the Frova introducer or other introducers and stylets, overinflation of the cuff) (Miñambres et al. 2009; Carbognani et al. 2004). The tracheal lesion mortality rate is extremely varying: in literature, we find case report of lesion 0.5 cm long that did not require any treatment (Sahin et al. 2012), several cases of pneumodiastinum with the necessity of surgical treatment (Kaneko 2006), and the worst cases that led to death. The surgical protocol of the Thoracic Surgical Unit of University of Campania “L. Vanvitelli” imposes several surgical techniques based on the entity of lesion: the conservative treatment in case of lesions inferior to 2 cm long, single-case evaluation if lesions are between 2 and 4 cm, and timely surgical treatment if mediastinic effusion, esophageal lesion, rupture of main bronchus or carina, and necessity of mechanical ventilation are present (Fiorelli et al. 2017). Recently, a study published by Cardillo has proposed a classification of tracheobronchial lesion based on depth and not length: level 1 in case of mucosa and sub mucosa laceration with pneumodiastinum and with or without subcutaneous emphysema; level 2 in case of tracheal lesion with the involvement of the lamina muscularis mucosae without mediastinic emphysema or without esophageal lesion; level 3A in case of complete lesion of the trachea with herniation of the esophagus or soft mediastinic tissues, without evidence of mediastinic or esophageal laceration; and level 3B in case of complete lesion of the trachea with mediastinic or esophageal laceration (Cardillo et al. 2010). In the case of levels 1 or 2, the conservative treatment is proposed; in the case of 3A, a conservative treatment in a high-qualified hospital is proposed, while in the case of level 3B, the surgical treatment is proposed (Cardillo et al. 2010). The Thoracic Surgery Unit of University of Campania “L. Vanvitelli” has been specializing in the endoscopic treatment of tracheobronchial lesions with fibrin glue. The inclusion criteria for being candidated to this technique are subcutaneous emphysema, cough and hemoptysis after an orotracheal intubation with evidence of tracheobronchial lesion inferior to 4 cm length after flexible diagnostic bronchoscopy, absence of mediastinitis or esophageal rupture, and hemodynamic stability.

From the anesthesiological point of view, the main problem is the airway management with special devices that will guarantee the breathing and oxygenation without worsening the tracheal rupture and interfering with thoracic surgeon’s practice. We have chosen three different kinds of airway devices: laryngeal mask, orotracheal tube positioned distally lesion, and one lung ventilation with a small size single tube. American Society of Anesthesiologist has defined the difficult airway as the clinical situation in which a conventionally trained anesthesiologist experiences difficulty with facemask ventilation of the upper airway, difficulty with tracheal intubation, or both. The difficulty in intubation is referred to intubation by classic laryngoscopy that requires more attempts so that requires more than 10 min. The difficult ventilations in the face mask is, instead, referred to the situation when it is not possible for the anesthesiologist to provide an efficient SpO_2_ with FiO_2_ 1 (Apfelbaum et al. 2013). The airway management during endoscopic treatment of tracheobronchial lesions so does not match with difficult airway criteria.

Another important aspect to evaluate before starting the procedure is the ventilation strategy during anesthesia conducted for the endoscopic treatment of tracheobronchial lesions. The effect of high pressure on the tracheobronchial lesions is very intuitive. In literature, there are several case reports about the negative impact of high pressure support during management of tracheobronchial rupture on outcome.

The publication of Farooqui is very interesting; it has demonstrated in a case report a correlation between the Peep level, even if very low (5 cm), and the hemodynamic impairment (Farooqui et al. 2014). The Peep level, in fact, correlates with the presence of pneumomediastinum and with a reduction of venous return and cardiac output.

The initial approach to ventilator settings is low tidal volume (VT) (6 ml/kg) and low PEEP (5 mmHg), to minimize expiratory air leak (Powner et al. 1981). The limited available clinical literature supports a plateau pressure (Ppl) less than 26 cmH for optimal compliance and gas exchange (Cheatham et al. 2006; Cinella et al. 2001). If we are not able to achieve the desired VT and Ppl, the switching to pressure-controlled mode with the same target Ppl has been shown to improve ventilation and reduce peak airway pressure (Litmanovitch et al. 1993). In patients with refractory respiratory acidosis, persistently elevated peak airway pressure, or oxygenation difficulties, salvage modes include high-frequency ventilation (HFV) (Mikita and Powell 2015). Some studies have found that in some cases of tracheobronchial lesion in otherwise healthy lungs, HFV improves oxygenation at lower mean airway pressures than conventional mechanical ventilation (Mikita and Powell 2015). The advantage of this method of ventilation is that it can allow effective gas transport without high airway pressure or depression of hemodynamic function and thus avoiding barotrauma or decreased cardiac output (Gillespie 1983). In our case series, we have approached the ventilation problems in case 1 by carrying out the procedure in spontaneous breathing, in case 2 by using a low Peep (5 mmHg), and in case 3 by the complete exclusion of bronchial lesion and one lung ventilation. The ventilation problem is strictly correlated to the airway management problem during the procedures. The limited available clinical literature supports that ventilation and airway management should prevent damage to the uninjured trachea, ensure adequate ventilation of the patient through the operative procedure, and finally facilitate the surgical repair of the affected bronchus (Mikita and Powell 2015). The limited available clinical literature supports that the spontaneous breathing in the case of conservative treatment of tracheal rupture is associated with the best outcome (Powner et al. 1981). So we have decided to conduct the case in LMA and in spontaneous breathing. When the spontaneous breathing is not possible, the airway management should aim to bridge the tracheal rupture with the tip and the cuff of the tube. The optimal method for protecting the repair is extubation, since spontaneous respiration places the least strain on the airways. Nevertheless, most multisystem trauma patients must remain intubated postoperatively. The limited available clinical literature supports the using of the lowest VT possible, the maintenance of Ppl under 26 mmHg, and considering modes that encourage spontaneous respirations during, such as intermittent mandatory ventilation (IMV) during postoperative ventilator management (Mikita and Powell 2015). In case 3, we decided to perform tracheostomy on the patient because we need to awaken her and to recover the spontaneous breathing but we did not consider the extubation safe. The flexible videolaryngoscopy, in fact, confirmed edema and hyperemia of the larynx, glottis, and subglottic region. Most of the literature on airway management deals with airway management in case of tracheal rupture and or conservative treatment or thoracotomy (Di Gaetano et al. 2014; Conti et al. 2007; Mitchell et al. 1993). Also, the jet ventilation has been tried especially in this kind of treatment (Powner et al. 1981). The innovative approach of the endoscopic treatment of tracheal rupture with fibrin glue has driven us to study these different kinds of airway management basing on the literature published. In case 3, we did not decide to use the double-lumen tube because of the size and location of rupture and so we chose a 5.5 size-reinforced endotracheal tube modifying the suggestions by Marquette (Conti et al. 2007). It should be better to use total intravenous anesthesia involving the use of analgesics, hypnotics, and anti-secretory agents as volatile anesthetic delivery cannot be guaranteed. The use of local anesthesia by topical lidocaine and inhalational techniques could precipitate coughing and so they should be avoided (Mitchell et al. 1993). The use of propofol/fentanyl to conduct anesthesia in spontaneous breathing was also described in another case report in a patient with tracheal dehiscence post-tracheal resection surgery. Kim et al. used a low-dose propofol infusion (25–50 μg/kg/min) and intermittent small bolus of fentanyl (25 μg) (Sanchit et al. 2016). The low doses of propofol and fentanyl do not inhibit hypoxic pulmonary vasoconstriction (Sanchit et al. 2016). An alternative as intravenous anesthetic agents should be ketamine and dexmedetomidine. Ketamine provides intense analgesia from the blockade of NMDA (*N*-methyl-d-aspartate) receptors with preservation of spontaneous breathing. The problem of its use in this setting is the increasing oral and airway secretions due to ketamine itself. There are recently several manuscript published on the use of dexmedetomidine in operative setting, but dexmedetomidine may be associated with hypotension and bradycardia and appropriate caution should be used (Sang et al. 2017; Parrillo et al. 2014; Lippmann et al. 1983; Niyogi et al. 2017; Sharma et al. 2017).

## Conclusion

The airway management in tracheal ruptures is difficult especially when we do not have to carry out an anesthesia for a thoracotomy but for a bronchoscopic approach. In these circumstances, the airway is partially filled with the bronchoscope itself and we could not use high pressure of ventilation in order to enhance the healing. The lack of specific guidelines on airway management in this kind of situation has led us to experiment these three different kinds of approach that we believe to be practical and useful. Using this kind of technique, we have obtained a protective ventilation on tracheal rupture, a rapid healing, and fast recovery of spontaneous breathing.

## Abbreviations

BMI: Body mass index
BP: Blood pressure
CT: Computerized axial tomography
HFV: High-frequency ventilation
HR: Heart rate
IMV: Intermittent mandatory ventilation
LMA: Laryngeal mask
MAP: Mean arterial pressure
PaO_2_: Partial pressure of oxygen
PCO_2_: Partial pressure of carbon dioxide
Peep: Positive end expiratory pressure
Ppl: Plateau pressure
SDML: Suspension direct microlaryngoscopy

## References

[CR1] Apfelbaum JL et al; American Society of Anesthesiologists Task Force on Management of the Difficult Airway. Practice guidelines for management of the difficult airway: an updated report by the American Society of Anesthesiologists Task Force on Management of the Difficult Airway Anesthesiology, 2013;118(2):251–270. 10.1097/ALN.0b013e31827773b2 23364566

[CR2] Carbognani P (2004). Management of postintubation membranous tracheal rupture. Ann Thorac Surg.

[CR3] Cardillo G (2010). Tracheal lacerations after endotracheal intubation: a proposed morphological classification to guide non-surgical treatment. Eur J Cardiothorac Surg.

[CR4] Cheatham ML (2006). Independent lung ventilation in the management of traumatic bronchopleural fistula. Am Surg.

[CR5] Cheney FW (1991). Adverse respiratory events infrequently leading to malpractice suits. A closed claims analysis. Anesthesiology.

[CR6] Cinella G (2001). Independent lung ventilation in patients with unilateral pulmonary contusion. Monitoring with compliance and EtCO(2). Intensive Care Med.

[CR7] Conti M (2007). Management of postintubation tracheobronchial ruptures. Ann Thorac Surg.

[CR8] Di Gaetano M (2014). Selective bilateral main stem bronchial intubation for the management of severe respiratory distress syndrome due to iatrogenic carinal perforation. Can J Anaesth.

[CR9] Domino KB (1999). Airway injury during anesthesia: a closed claims analysis. Anesthesiology.

[CR10] Farooqui AM (2014). Unusual case of acute tracheal injury complicated by application of positive end expiratory pressure (PEEP). BMJ Case Rep.

[CR11] Fiorelli A (2017). Endoscopic treatment with fibrin glue of post-intubation tracheal laceration. J Vis Surg.

[CR12] Gil T (2016). Iatrogenic injuries to the trachea and main bronchi. Kardiochir Torakochirurgia Pol.

[CR13] Gillespie DJ (1983). High-frequency ventilation: a new concept in mechanical ventilation. Mayo Clin Proc.

[CR14] Harenberg T (2010). Distal trachea and bronchial large lesions and suture reinforcement with Polyglicol Acid (PGA) patch. First clinical experience. G Chir.

[CR15] Iwanczuk W (2008). Iatrogenic tracheal rupture, tension pneumothorax and cardiac arrest. Anestezjol Intens Ter.

[CR16] Kaneko Y (2006). Subcutaneous emphysema and pneumomediastinum after translaryngeal intubation: tracheal perforation due to unsuccessful fiberoptic tracheal intubation. J Clin Anesth.

[CR17] Lippmann M (1983). Sequential cardiorespiratory patterns of anesthetic induction with ketamine in critically ill patients. Crit Care Med.

[CR18] Litmanovitch M (1993). Persistent bronchopleural fistula in a patient with adult respiratory distress syndrome. Chest.

[CR19] Mikita JA, Powell D (2015). Ventilation for tracheal disruption and bronchopleural fistula in Buckenmaier C. Combat anesthesia: the first 24 hours.

[CR20] Miñambres E (2009). Tracheal rupture after endotracheal intubation: a literature systematic review. Eur J Cardiothorac Surg.

[CR21] Mitchell JB (1993). The management of tracheal rupture using bilateral bronchial intubation. Anaesthesia.

[CR22] Niyogi S (2017). Efficacy of intravenous dexmedetomidine on patient’s satisfaction, comfort and sedation during awake fibreoptic intubation in patients with cervical spondylotic myelopathy poted for elective cervical fixation. Indian J Anaesth.

[CR23] Parrillo JE (2014). Critical Care Medicine: principles of diagnosis and management in the adult.

[CR24] Powner DJ (1981). Ventilatory management of life-threatening bronchopleural fistulae. Crit Care Med.

[CR25] Sahin M (2012). Case reports: iatrogenic bronchial rupture following the use of endotracheal tube introducers. Can J Anaesth.

[CR26] Sanchit A (2016). Practical anesthetic considerations in patients undergoing tracheobronchial surgeries: a clinical review of current literature. J Thorac Dis.

[CR27] Sang K (2017). Anesthetic management of a patient with tracheal dehiscence post–tracheal resection surgery. Semin Cardiothorac Vasc Anest.

[CR28] Schneider T (2009). Incidence and treatment modalities of tracheobronchial injuries in Germany. Interact Cardiovasc Thorac Surg.

[CR29] Sharma J (2017). Awake orotracheal fibre-optic intubation: comparison of two different doses of dexmedetomidine on intubation conditions in patients undergoing cervical spine surgery. Indian J Anaesth.

